# Comparative transcriptome analysis of major lodging resistant factors in hulless barley

**DOI:** 10.3389/fpls.2023.1230792

**Published:** 2023-10-13

**Authors:** Yixiong Bai, Xiaohong Zhao, Xiaohua Yao, Youhua Yao, Xin Li, Lu Hou, Likun An, Kunlun Wu, Zhonghua Wang

**Affiliations:** ^1^ Qinghai University, Qinghai Academy of Agricultural and Forestry Sciences, Qinghai Key Laboratory of Hulless Barley Genetics and Breeding, Xining, Qinghai, China; ^2^ State Key Laboratory of Crop Stress Biology for Arid Areas, College of Agronomy, Northwest A&F University, Shaanxi, China; ^3^ Good Agricultural Practices Research Center of Traditional, Chongqing Institute of Medicinal Plant Cultivation, Chongqing, China

**Keywords:** hulless barley, lignin, stem plant lodging resistance, RNA-Seq, transcription factors

## Abstract

Hulless barley (Hordeum vulgare L. var. nudum Hook. f.), belonging to the genus Gramineae, has high and steady output and thus considered as a principal food crop by Tibetan people. Hulless barley grain can be used for food, brewing, and functional health product development, while its straw serves as an essential supplementary forage and is a crucial cereal crop. Lodging can reduce the yield and quality of barley grain and straw, and it hinders mechanical harvesting. It is a significant factor affecting high and stable yields of barley. Unlike other Poaceae plants (such as rice, wheat), hulless barley is mainly grown in high-altitude regions, where it is susceptible to low temperatures, strong winds, and heavy rainfall. As a result, its stem lodging resistance is relatively weak, making it prone to lodging during the growth period. In this study, we observed that the lignin concentration and the contents of lignin monomers (H, S, and G), and neutral detergent fibre of the lodging-resistant variety Kunlun14 were substantially greater than those of the lodging-sensitive variety Menyuanlianglan. We performed the weighted gene co-expression network analysis (WGCNA) and Short Time-series Expression Miner (STEM) analysis of both the lodging-resistant and lodging-sensitive varieties. Through transcriptome sequencing analysis at different developmental stages, combined with the previously annotated genes related to lodging resistance, a total of 72 DEGs were identified. Among these DEGs, 17 genes were related to lignin, cellulose, and hemicellulose synthesis or regulation, including five transcription factors about NAC, MYB and WRKY. Our results provide a basis for further exploring the molecular mechanism of stem lodging resistance in hulless barley and provide valuable gene resources for stem lodging resistance molecular breeding.

## 1 Introduction

Barley is one of the most primitive domesticated crops that is widely used in winemaking, as food, and for other applications(Badr et al., 2000; Ullrich, 2011). Qingke, also known as hulless barley, has long been a staple diet in the Qinghai-Tibet Plateau. Lodging is one of the most severe problems encountered by Gramineae crops that not only diminishes the yield but also deteriorates the quality. Its incidence has been reported in wheat (Wang et al., 2006; Foulkes et al., 2011), rice (Unan et al., 2013), and barley (Berry et al., 2004). One of the most significant targets of grain breeding is to obtain lodging-resistant varieties. The first green revolution aimed to convert tall stalks into small stalks (Khush, 1999). One of the most critical variables determining crop lodging resistance is stem characteristics. Lodging resistance is directly related to the morphological structure and physiological features of stems. Improving stem mechanical strength can greatly increase crop lodging resistance (Shah et al., 2017). Studies have shown that lignin, cellulose, and hemicellulose are associated with increased stem mechanical strength and lodging resistance (Lewis and Yamamoto, 1990; Cosgrove, 2005). Therefore, enhancing the synthesis of these three compounds is essential to enhance plant lodging resistance and stem hardness. Additionally, studies have demonstrated that lignin and lignin-like compounds, along with other cell wall components, provide resistance to diseases, insects, low temperature, and other biotic and abiotic stresses (Moura et al., 2010; Cesarino, 2019). Barley is an important economic crop in the food and beer industries, and its abundant lignocellulosic residues can be converted into biofuels and biochemicals. Despite the recalcitrance of lignocellulose providing mechanical strength to sustain plant lodging resistance in biomass production, it also leads to the costly process of biofuel production (Zhang et al., 2023).

Lignin is polymerised from the lignin monomer and synthesised through different pathways such as the shikimic acid pathway, phenylpropane pathway, and specific lignin synthesis pathway. The intracellular lignin monomers are transported to the cell wall and catalytically polymerised to form lignin (Vanholme et al., 2010; Vanholme et al., 2013). According to the monomer type, lignin can be divided into syringyl lignin (S-lignin), guajacyl lignin (G-lignin), and hydroxyphenyl lignin (Zhao, 2016). Furthermore, caffeine-based C-type lignin was discovered in Cleome hassleriana and Cactaceae plants (Chen et al., 2013; Zhuo et al., 2019). Several enzymes such as phenylalanine ammonia-lyase (PAL), cinnamate 4-hydroxylase (C4H), 4-coumarate: CoA ligase (4CL), p-coumarate 3-hydroxylase (C3H), cinnamate O-methyltransferase (COMT), caffeoyl-CoA O-methyltransferase (CCoAMOT), ferulate 5-hydroxylase (F5H), cinnamyl-CoA reductase (CCR), and cinnamyl alcohol dehydrogenase (CAD) are involved in lignin biosynthesis (Vanholme et al., 2010).

Cellulose is a major component of plant cell walls that employs uridine diphosphate-glucose (UDP-glucose) as a substrate and relies on the cellulose synthase gene (*CesA*), which catalyse the synthesis of glucan chains, eventually resulting in the formation of cellulose microfilaments. The research by Li et al. indicates that CESA9 mutations may influence the integrity of the CESA4/7/9 complex, resulting in proteasomal degradation of CESA proteins in low degree of polymerization (DP) cellulose biosynthesis. Consequently, this reduces cellulose crystallinity (CrI) and affects the lodging resistance properties of rice (Li et al., 2017). This catalytic process is highly complex and involves enzymes such as sucrose synthase (SUSY), cytosolic invertase (CINV), and UDP-glucose pyrophosphorylase (Fujii et al., 2010; Fan et al., 2017; Rende et al., 2017; Barnes and Anderson, 2018).

Hemicellulose is the key component of secondary plant walls and is composed of various monosaccharides such as xylose, galactose, glucose, mannose, and primarily xylan (Scheller and Ulvskov, 2010). The glycosyltransferase complex is primarily involved in xylan synthesis. Additionally, the xylan synthase complex exhibits transferase activity, which induces the formation of 4-glycosidic bonds (Ren et al., 2014; Zhao et al., 2018) in the main xylitol chain. Glycosyltransferases (GTs) are the primary enzymes involved in xylan production. Additionally, the research conducted by Fan et al. demonstrates that the transgenic rice plants with the *OsEXTL* gene exhibit a significant increase in secondary wall thickening and higher cellulose levels in the mature stems. This leads to a significant enhancement in the mechanical strength (tensile and compressive strength) of the transgenic rice stems, resulting in a substantial improvement in their lodging resistance capability (Fan et al., 2018). A hemicellulose-arabinose is detected as a major factor that potentially negatively affects cellulose Crystallinity Index (CrI) through its interaction with b-1,4-glucan. Simultaneous overexpression of *GH9B* and *XAT* genes in rice can reduce cellulose CrI and increase arabinose levels, thereby modifying the cell wall to enhance the lodging resistance properties of rice plants (Li et al., 2015).

The primary transcription factors that regulate the synthesis of lignin, cellulose, and hemicellulose include NAC, MYB, WRKY, and zinc finger proteins (Wen et al., 2023; Xue et al., 2023; Zhao et al., 2023). MYB can directly control genes involved in the lignin production pathway. In pears, MYB169 binds to the promoters of lignin biosynthesis genes, such as *C3H1*, *CCR1*, *CCOMT2*, *CAD*, *4CL1*, *4CL2*, *HCT2*, and *LAC18*, to activate AC elements and thus increases the lignin content (Xue et al., 2019; Zhong et al., 2019; Geng et al., 2020). In maize, NAC enhances *CesA* gene expression by boosting MYB transcription, thereby enhancing cellulose synthesis (Xiao et al., 2018). NAC may promote the expression of xylan-related genes in Betula platyphylla, a woody plant, thus stimulating hemicellulose synthesis (Hu et al., 2019). WRKY influences lignin production in alfalfa by modulating *COMT* (Gallego-Giraldo et al., 2016). Zinc finger protein inhibits lignin production in rice by modulating *CAD2* and *CAD3* (Huang et al., 2018). Furthermore, the NAC-MYB-based lignin synthesis regulation model NAC-MYB-GRN (Ohtani and Demura, 2019), MBW (MYB-bHLH-WD40) complex regulates lignin synthesis by regulating the phenylpropane pathway, and plant hormones such as auxin, ethylene, gibberellin, and monopodium lactone are involved in lignin production (Dong and Lin, 2020).

In the present study, we screened 17 genes related to lignin, cellulose, and hemicellulose synthesis through the WGCNA and STEM analysis of the lodging-resistant variety Kunlun14 and the lodging-sensitive variety Menyuanlianglan at different developmental stages. In addition, we explored the mechanism of lodging resistance at the transcriptional level. The identified genes include five transcription factors, laying the groundwork for future research on lignin, cellulose, and hemicellulose synthesis and regulation in hulless barley, which may improve the breeding process.

## 2 Materials and methods

### 2.1 Materials

**Figure 1:**
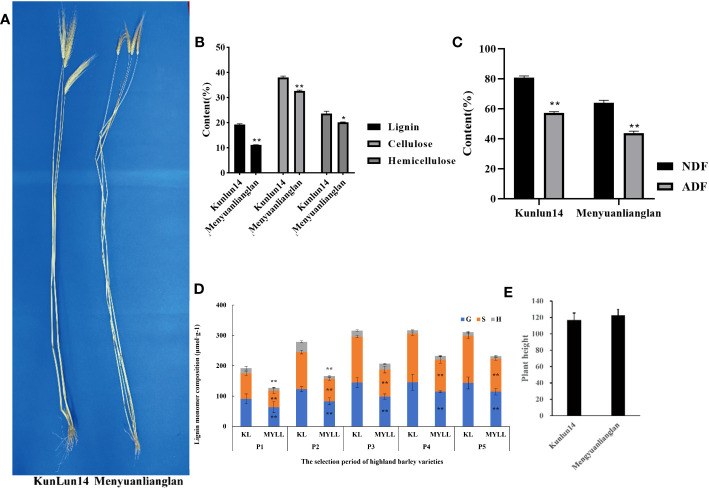
**(A)** Comparison between Kunlun14 and Mengyuanlianglan; **(B)** Lignin, cellulose, and hemicellulose contents in hulless barley stalks of the two varieties; **(C)** Neutral detergent fibre (NDF) and the acid detergent fibre (ADF) contents in hulless barley stalks of the two varieties. **(D)** Lignin monomer contents in hulless barley stalks of the two varieties, **(E)** Plant height in hulless barley stalks of the two varieties. *; P<0.05; **: P<0.01.

**Figure 1A** shows the stable lodging-resistant Qingke variety Kunlun14 (KL) and the seriously lodging Qingke variety Menyuanlianglan (MYLL). All materials were planted in April at the Academy of Agriculture and Forestry, Qinghai University (36°43′N, 101°45′E) and harvested in August. The samples from these varieties were collected at the jointing stage, early stem formation stage (P1), stem formation stage (P2), late flowering stage (P3), filling stage (P4), and mature stage (P5). RNA extraction and transcriptome sequencing were performed during these five stages.

### 2.2 Lignin content determination

The lignin content in the stem of hulless barley was determined using the methods described by Vanholme et al. (Vanholme et al., 2013), Zhou et al. (Zhou et al., 2009) and Harman-Ware et al. (Harman-Ware et al., 2016). The lignin composition in the stem of hulless barley was determined according to the method specified by Muhammad et al. (Muhammad et al., 2020). The stems of Kunlun14 and Menyuanlianglan were dried in an oven and then crushed into powder. Accurately weighed 2 mg of the sample was taken and mixed with 25% of new bromoacetyl solution prepared with glacial acetic acid for extraction. The test tube containing the mixture was placed in a water bath at 50°C for 3 h. At the end of the reaction, the test tube was placed on an ice bath to allow it to cool rapidly to the room temperature. Then, 800 μL of sodium hydroxide solution (2 mol·L^-1^) and 140 μL of freshly prepared hydroxylamine hydrochloride solution (0.5 mol·L^-1^) were added, and the mixture was shaken well. The mixture was then transferred to a 15-mL calibration tube, and the volume was made up to 15 mL with glacial acetic acid. The absorbance of the mixture was determined using an ultraviolet spectrophotometer at a 280-nm wavelength to determine the percentage content of lignin.

### 2.3 RNA extraction, Illumina library construction, and sequencing

Total RNA was extracted using the TRIzol reagent kit (Invitrogen, Carlsbad, CA, USA), according to the manufacturer’s protocol. RNA quality was assessed using an Agilent 2100 Bioanalyzer (Agilent Technologies, Palo Alto, CA, USA) and subjected to RNase-free agarose gel electrophoresis. After extracting the total RNA, eukaryotic mRNA was enriched by oligo(dT) beads, whereas prokaryotic mRNA was enriched by removing rRNA using the Ribo-ZeroTM Magnetic Kit (Epicentre, Madison, WI, USA). Then, the enriched mRNA was fragmented into short fragments by using fragmentation buffer and reverse transcribed into cDNA with random primers. Second strand of cDNA was synthesised by DNA polymerase I, RNase H, dNTP, and buffer. Then, the cDNA fragments were purified using the QiaQuick PCR extraction kit (Qiagen, Venlo, The Netherlands), end repaired, polyadenylated, and ligated to Illumina sequencing adapters. The ligation products of desired size were selected by agarose gel electrophoresis, PCR amplified, and sequenced using Illumina HiSeq4000 (Allwegene Technologies Corporation, Beijing, China).

### 2.4 Transcriptome data processing

Reads obtained from the sequencing machines include raw reads containing adapters or low-quality bases, which can affect the assembly and analysis. Thus, to obtain high-quality clean reads, the reads were further filtered by fastp (version 0.18.0) (Chen et al., 2018).

Sample high-quality reads were aligned to the Qingke reference genome (ftp://ftp.ensemblgenomes.org/pub/plants/release-44/fasta/hordeum_vulgare/dna/) by using hisat2 (Kim et al., 2015). The aligned reads were subsequently used to create a reference annotation-based transcript assembly, which was used to identify uniquely aligned reads in each sample. The results were subjected to “HTSeq” (Anders et al., 2015) to obtain the read count of all samples. Differences in the gene expression were determined using “DESeq” (Anders and Huber, 2010). Gene Ontology (GO) enrichment analysis (including biological process, cellular component, and molecular function) of differentially expressed genes (DEGs) across the samples was performed using the GOseq (Young et al., 2010).

For each transcription region, a FPKM (fragment per kilobase of transcript per million mapped reads) value was calculated to quantify its expression level and variations by using StringTie software (Pertea et al., 2015).

Based on the normalized expression data (log2 transformed), all DEGs with similar expression trends were clustered using STEM (Ernst and Bar-Joseph, 2006) to obtain gene expression profiles related to the duration of hypoxic exposure. The threshold cluster number and cor-relation coefficient were set to 20 and 0.7, respectively, and P <0.05 was used as the threshold for significance.

### 2.5 Construction of gene co-expression networks

Co-expression networks were constructed using the WGCNA (v. 1.47) package in R software (Langfelder and Horvath, 2008). Overall, 13011 genes expressed in the all samples were included in the WGCNA workflow of Kunlun 14 and Munyuanlianglan, respectively. The module eigenvectors were calculated, and similar modules were merged, which yielded 27 gene co-expression modules for both varieties. To explore the biological meaning of each model, GO enrichment analysis was performed using a free online platform, OmicShare (www.omicshare.com/tools).

### 2.6 Quantitative real-time polymerase chain reaction validation

The expression of 5 randomly selected target genes was validated by quantitative real-time polymerase chain reaction (qRT-PCR). Reverse transcription was performed using the M-MLV Reverse Transcriptase Kit (Invitrogen). Real-time PCR was conducted on the ViiA7 Real-time PCR system (Applied Biosystems, Foster City, CA, USA) by using the FastStart Universal SYBR Green Master (ROX) (Roche). *ACTIN* gene was used as a reference, and each sample was assessed in three technical replicates. The cycling parameters were: 95°C for 30 s (1 cycle); 95°C for 5 s, 60°C for 30 s (40 cycles); 95°C for 5 s, 60°C for 1 min (1 cycle); and 40°C for 5 s (1 cycle). Gene expression was calculated using the 2^–ΔΔCt^ method, with the Ct value being the average of three biological replicates with three technical replicates.

## 3 Results

### 3.1 Lignin and cellulose levels of mature stems varied in two hulless barley varieties

Lignin and cellulose are the two major elements that control stalk strength. Therefore, the lignin and cellulose contents of the lodging-resistant and -sensitive varieties were assessed. The contents of lignin and lignin monomers (H, S, and G) in the lodging-resistant variety Kunlun14 were found to be much greater than those in the lodging-sensitive variety Menyuanlianglan (**Figures 1B**, **D**). In terms of the fibre content, Kunlun14 exhibited a substantially higher neutral detergent fibre (NDF) content than Menyuanlianglan; however, the two varieties exhibited no significant difference in the acid detergent fibre (ADF) content (**Figure 1C**). Menyuanlianglan has a greater plant height than Kunlun14 and a lodging rate of up to 92%. However, the difference in plant height between the two cultivars was nonsignificant (p = 0.09) (**Figure 1E**).

### 3.2 Numbers and expression levels and enrichment analysis of DEGs

**Figure 2:**
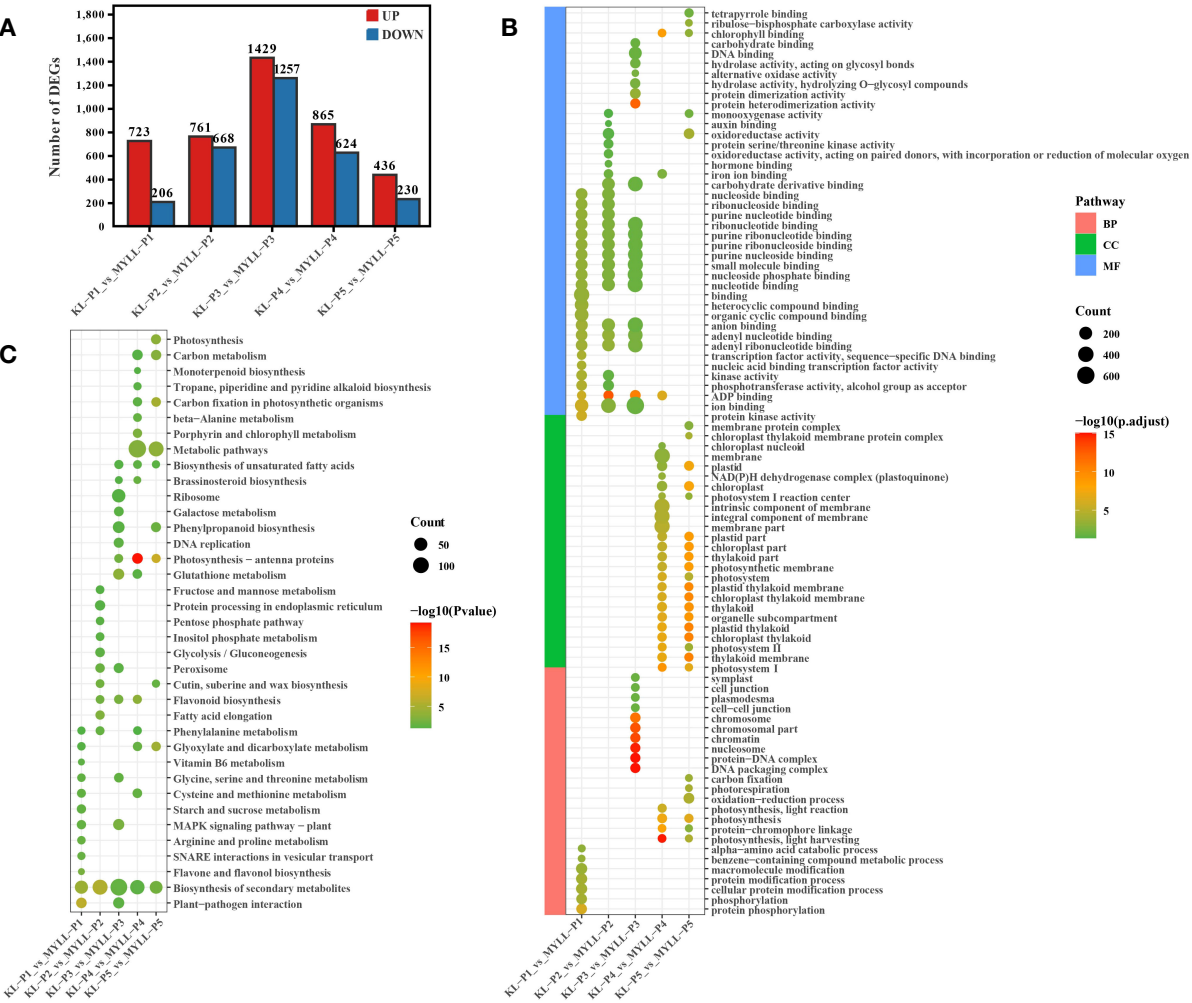
**(A)** The number of DEGs among different varieties; The GO **(B)** and KEGG **(C)** enrichment analysis of DEGs.

The |log2Foldchang|>1 and adjusted *P* value < 0.05 were considered as the gold standard to identify DEGs. When the two varieties were compared at different stages, KL-P1 had 723 genes upregulated and 206 downregulated relative to MYLL-P1, 761 upregulated and 668 downregulated in P2, 1429 upregulated and 1257 downregulated in P3, 865 upregulated and 624 downregulated in P4, 436 upregulated and 230 downregulated in P5 (**Figure 2A**). The number of differentially expressed genes during the entire development period showed a normal distribution, which indicates that as the development period progresses, the number of genes that may be involved in lignin synthesis increases first and then decreases, which is consistent with the growth law of plants. The maximum number of DEGs was identified at the P3 stage (late flowering stage) with 2686, suggesting that these genes could be biologically important, may be involved in the synthesis of lignin, cellulose, and hemicellulose in highland barley.

Gene ontology (GO) annotations can be classified into three distinct categories, molecular function (MF), biological process (BP), and cellular component (CC). To gain insights into potential gene function, we performed GO analyses (p-value < 0.05) on the sequence data from the five experimental groups, the top 30 GOs were selected for visualization, and the whole information of the GO results were shown in **Figure 2B**.

In the P1 group, the most enriched biological processes were protein phosphorylation, protein modification process and macromolecule modification. In molecular function terms were enriched in nucleic acid related metabolic processes (**Figure 2B**).

In the P2 group, the most enriched biological processes were enriched in only one type of molecular function. In this type, DEGs were not only enriched in nucleic acid related metabolic processes, but enriched oxidoreductase activity, oxidoreductase activity (**Figure 2B**).

In the P3 group, the most enriched biological processes were enriched DNA packaging complex, protein-DNA complex, cell-cell junction. In molecular function terms were not only enriched in nucleic acid related metabolic processes, but also enriched carbohydrate binding, hydrolase activity (acting on glycosyl bonds), alternative oxidase activity (**Figure 2B**).

In the P4 and P5 group, the DEGs were enriched in cellular component ontology. In this ontology, the DEGs were mainly enriched photosynthetic membrane, chloroplast thylakoid membrane, integral component of membrane. In addition, DEGs also were enriched photosynthesis, protein-chromophore linage in biological processes ontology, and chlorophyl II binding in molecular function ontology (**Figure 2B**).

To better understand the functions of the DEGs, we performed KEGG pathway analysis. In the P1 group, the DEGs were mainly enriched plant-pathogen interaction, biosynthesis of secondary metabolites, flavone and flavonol biosynthesis, phenylalanine metabolism, starch and sucrose metabolism. In addition, the significant pathways also included amino acid metabolism, such as arginine, proline, glycine, serine, threonine, cysteine and methionine (**Figure 2C**).

In the P2 group, the DEGs were mainly enriched biosynthesis of secondary metabolites, glycolysis/gluconeogenesis, phenylalanine metabolism and flavonoid biosynthesis (**Figure 2C**).

The enriched pathway of the P3 group were biosynthesis of secondary metabolites, flavonoid biosynthesis, glutathione metabolism, phenylpropanoid biosynthesis, galactose metabolism. Metabolic pathways were the most enriched in the P4 and P5 group, additionally, biosynthesis of secondary metabolites, biosynthesis of unsaturated fatty acids, glyoxylate and dicarboxylate metabolism, carbon metabolism and photosynthesis (**Figure 2C**).

### 3.3 STEM analysis of DEGs

To further analyse the differentially expressed genes associated with important biochemical processes, we performed the Short Time-series Expression Miner (STEM) over five time points and obtained 4 and 5 theoretical gene expression tendencies in Kunlun14 and Menyuanlianglan.

**Figure 3:**
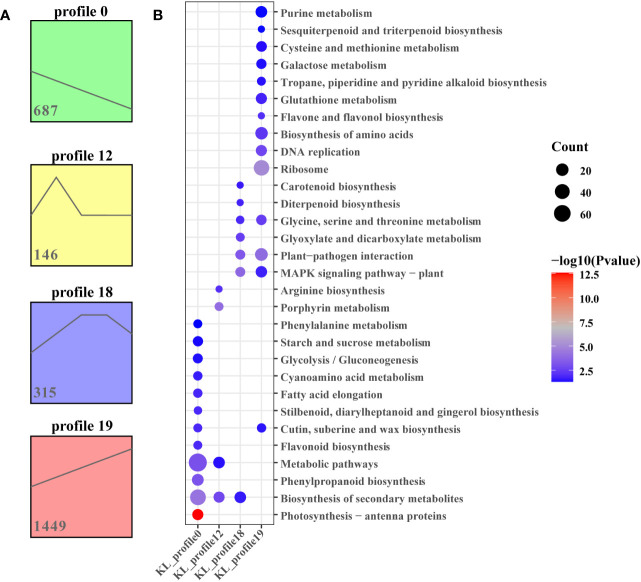
STEM analysis of all DEGs. **(A)** Four expression profiles at five stages in Kunlun14; **(B)** The KEGG enrichment of in profiles 0, 12, 18 and 19.

As determined using STEM, four significant (P < 0.05) gene expression profiles (profiles 0, 12, 18, and 19) involving 2597 DEGs related to development periods of Kunlun14 were screened. Profiles 0 and 19 exhibited clear gene expression trends, while the trends in profiles 12 and 18 were not clear (**Figure 3A**). At 5 different development periods of Kunlun14, the expression levels of 687 DEGs in profile 0 (P = 0) continued to decrease and 1449 DEGs in profile 19 continued to increase (**Figure 3A**). Through KEGG enrichment analysis, significant enrichments were found in pathway such as “biosynthesis of secondary metabolites, metabolic pathways, phenylpropanoid biosynthesis, phenylalanine metabolic, starch and sucrose metabolism” in profile 0. In profile 19, the DEGs were significant enriched biosynthesis of amino acids, flavone and flavonol biosynthesis, sesquiterpenoid and triterpenoid biosynthesis. Although the 146 DEGs in profile 12 (P = 1.8e-5) were up-regulated at P1-P2 and then down-regulated at P2-P3, and then remained unchanged (**Figure 2A**). As demonstrated in **Figure 2A**, the gene expression levels in profile 18 (P =3.8e-5, 315 DEGs) were generally up-regulated at P1-P3 stage and remained unchanged at P3-P4, and then down-regulated at P4-P5. In addition, the results of KEGG enrichment analyses of genes in profiles 12 and 18 were enriched to arginine biosynthesis, MAPK signalling pathway-plant, glycine, serine and threonine metabolism.

**Figure 4:**
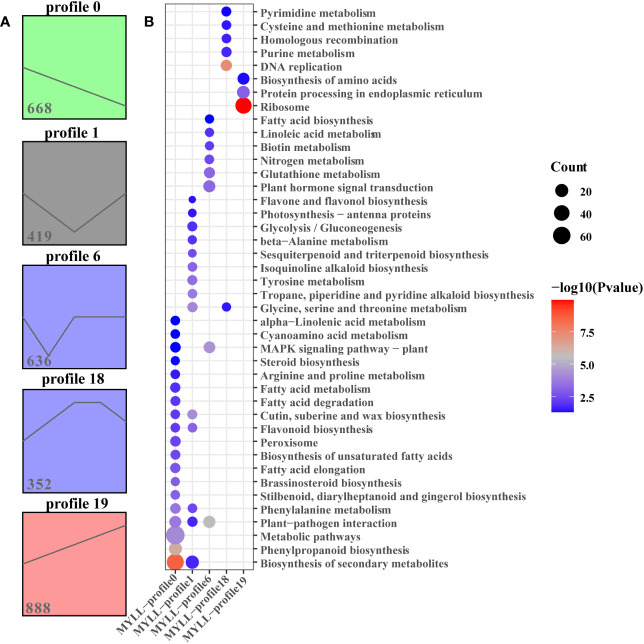
STEM analysis of all DEGs. **(A)** Four expression profles at five stages in Menyuanlianglan; **(B)** The KEGG enrichment of in profiles 0, 1, 6, 18 and 19.

As determined using STEM, five significant (P < 0.05) gene expression profiles (profiles 0, 6, 12, 18, and 19) involving 2963 DEGs related to development periods of Kunlun14 were screened. Profiles 0, 1 and 19 exhibited clear gene expression trends, while the trends in profiles 12 and 18 were not clear (**Figure 4A**). At 5 different development periods of Kunlun14, the expression levels of 668 DEGs in profile 0 (P = 4.9e-130) continued to decrease and 888 DEGs in profile 19 (P = 2.2e-262) continued to increase (**Figure 4A**). The 419 DEGs in profile 1 (P = 0.01) were down-regulated at P1-P3 and then up-regulated at P3-P5 (**Figure 4A**). Through KEGG enrichment analysis, significant enrichments were found in pathway such as “biosynthesis of secondary metabolites, metabolic pathways, phenylpropanoid biosynthesis, phenylalanine metabolic, and some relative to fatty acid metabolism” in profile 0. In profile 19, the DEGs were significant enriched in the metabolism related to amino acids (such as glycine, serine, threonine, alanine and tyrosine), flavone and flavonol biosynthesis, sesquiterpenoid and triterpenoid biosynthesis. In contrast to the other two profiles, in profile 1, significant enrichment was observed in pathways such as “plant hormone signal transduction, nitrogen metabolism, biotin metabolism and glutathione metabolism”. Although the 146 DEGs in profile 6 (P = 1.9e-117) were down-regulated at P1-P2 and then up-regulated at P2-P3, and then remained unchanged. (**Figure 2A**). As demonstrated in **Figure 2A**, the gene expression levels in profile 18 (P =4.1e-11, 352 DEGs) were generally up-regulated at P1-P3 stage and remained unchanged at P3-P4, and then down-regulated at P4-P5. In addition, the results of KEGG enrichment analyses of genes in profiles 6 and 18 were enriched to ribosome, protein processing in endoplasmic reticulum, DNA replication, purine metabolism, pyrimidine metabolism.

### 3.4 DEGs and potential lodging resistance-related genes

There are significant differences in the content of lignin, cellulose and hemicellulose between Kunlun14 and Menyuanlianglan varieties. In actual production, Kunlun14 has stronger lodging resistance than Menyuanlianglan. Therefore, we analyzed the genes that may be involved in lignin, cellulose and hemicellulose synthesis and metabolism among them.

**Figure 5:**
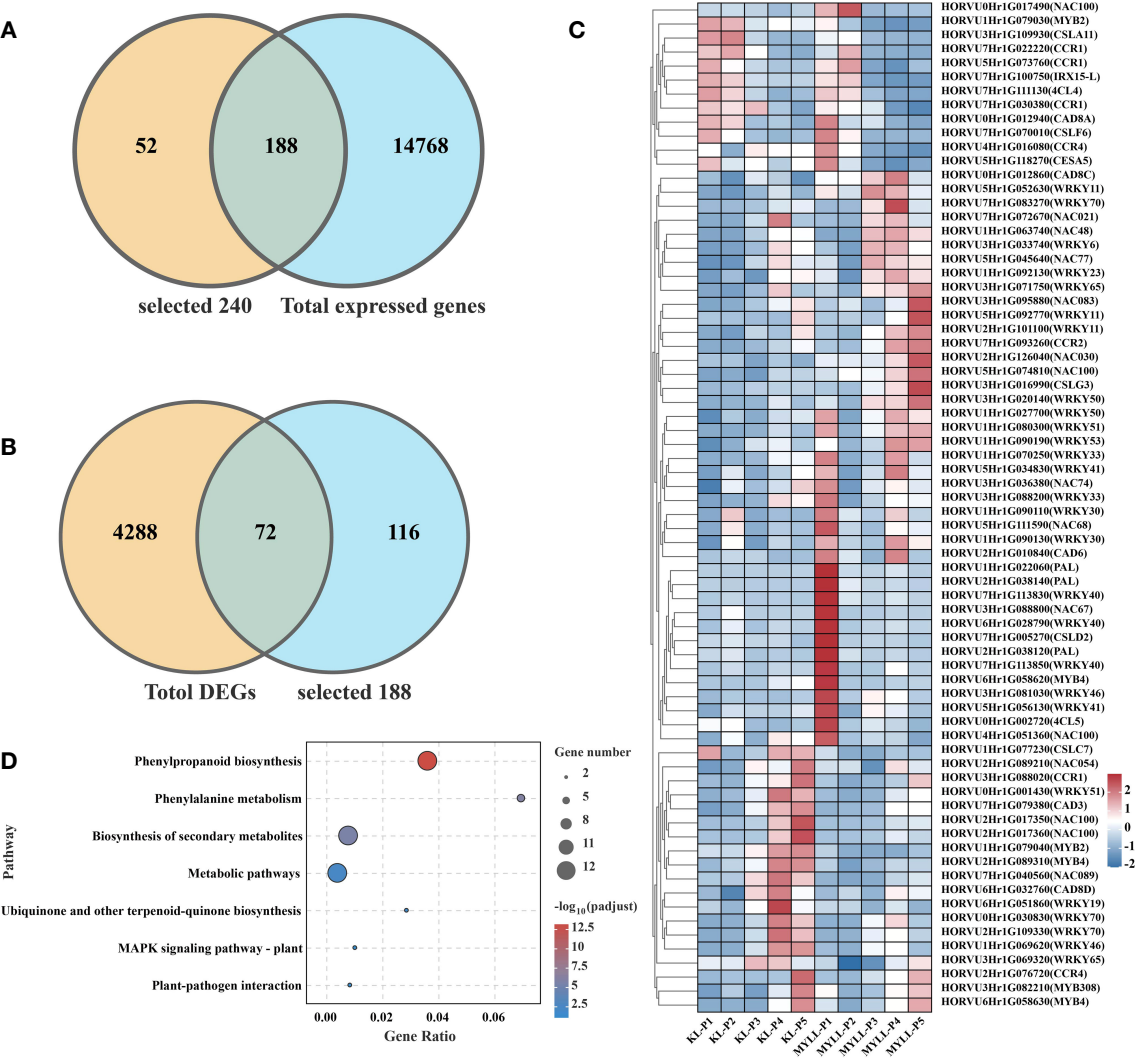
**(A)** Gene annotation and Venn map of DEGs; **(B)** Venn diagram of DEGs and lignin synthesis-related genes; **(C)** Heatmap of DEGs in different varieties; **(D)** The KEGG enrichment of 72 DEGs is related to lignin synthesis and regulation-related genes.

First, we discovered 14,956 expressed genes between Kunlun14 and Menyuanlianglan by an analysis of transcriptomes during different developmental stages. Based on the findings of genome annotation and transcriptome analysis of hulless barley, we identified a total of 240 potential lodging resistance-related genes. Among these genes, 188 were observed to be differently expressed (**Figure 5A**). The expression of DEGs of the two varieties was compared, which indicated that the number of DEGs reached its peak at the late flowering stage (P3), and a total of 4360 DEGs were obtained in five stages, which were combined with the previously annotated genes related to lodging resistance to yield a total of 72 DEGs (**Figure 5B**). Among these 72 DEGs, many are involved in the synthesis of lignin, cellulose and hemicellulose compounds, as well as transcription factors that may be involved in the regulation of lignin, cellulose and hemicellulose, such as PAL, CCR, NAC, etc. Among these genes, some genes, such as *CSLA11* (*HORVU3Hr1G109930*) and *CCR1* (*HORVU7Hr1G022220*, *HORVU7Hr1G030380*), are expressed at higher levels in the P1 and P2 stages of Kunlun14 than in Menyuanlianglan. Some transcription factors, such as *NAC054* (*HORVU2Hr1G089210*), *WRKY51* (*HORVU0Hr1G001430*), *NAC100* (*HORVU2Hr1G017350*, *HORVU2Hr1G017360*), *WRKY19* (*HORVU6Hr1G051860*), *MYB2* (*HORVU1Hr1G079040*), are expressed at higher levels in the P4 and P5 stages of Kunlun14 than in Menyuanlianglan (**Figure 5C**). The KEGG enrichment analysis of these 72 genes revealed significant enrichment in pathways such as “biosynthesis of secondary metabolites, metabolic pathways, phenylpropanoid biosynthesis, phenylalanine metabolic”, which are involved in the biosynthesis of lignin, cellulose and hemicellulose compounds that originate from the phenylalanine pathway, and some synthesis pathways overlap with the synthesis of flavonoids (**Figure 5D**).

### 3.5 DEGs of different developmental stages in Kunlun14 and Menyuanlianglan

**Figure 6:**
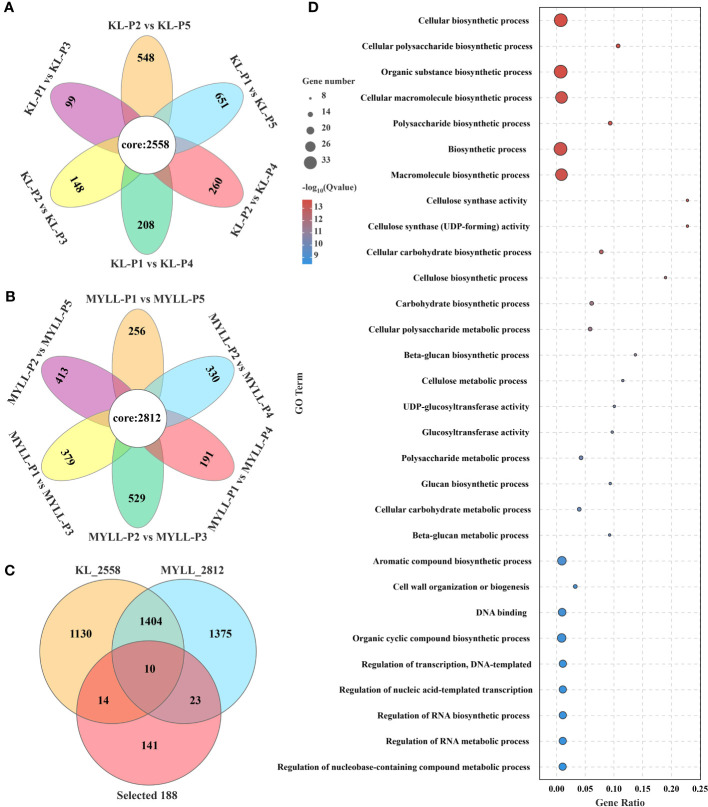
Venn map of DEGs in different developmental stages of lodging variety **(A)** and lodging-resistant variety **(B)**; **(C)** Venn map of DEGs in the two varieties; **(D)** GO enrichment analysis of 47 DEGs.

To better analyse the lodging-resistant genes in Kunlun14, we compared the different developmental times of Kunlun14 and Menyuanlianglan two varieties. In different developmental stages (P1 *vs*. P2, P1 *vs*. P3, P1 *vs*. P4, P1 *vs*. P5), differential genes were shown with Venn diagram, 2558 DEGs were observed in the lodging-resistant variety Kunlun14 (**Figure 6A**), whereas 2812 DEGs were observed in the lodging variety Menyuanlianglan (**Figure 6B**).

In the Kunlun 14 and Menyuanlianglan groups, only 24 and 33 DEGs linked to lignin production, respectively, were identified (**Figure 6C**). The expression of 47 DEGs between the two types is demonstrated in **Figure 6C**. According to gene annotation results, 26 genes were involved in the lignin, cellulose, and hemicellulose biosynthesis pathways (total 106 genes), with 21 belonging to the MYB, NAC, and WRKY transcription factor families, which may regulate lignin biosynthesis. Further GO enrichment analysis revealed that these 47 genes are significantly enriched in GO terms such as “cellulose synthase activity, cellulose biosynthetic process, cell wall organization or biogenesis, polysaccharide biosynthetic process, etc” (**Figure 6D**). The GO enrichment analysis results indicated the association of the identified genes with stem strength.

**Figure 7:**
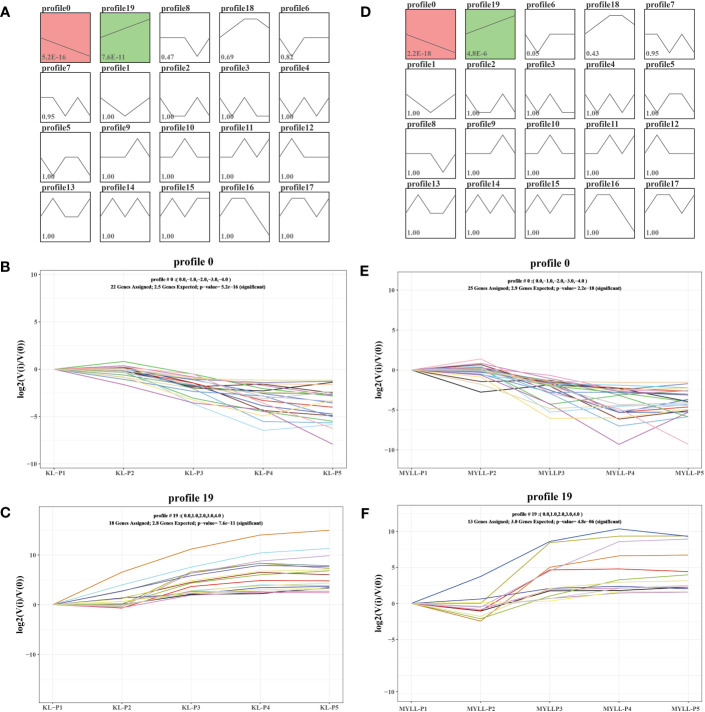
STEM analysis of 47. Twenty expression profiles in Kunlun14 **(A)** and Menyuanlianglan **(D)**; Expression trends for genes in profile 0 in Kunlun14 **(B)** and Menyuanlianglan **(E)**; Expression trends for genes in profile 19 in Kunlun14 **(C)** and Menyuanlianglan **(F)**.

To further analyse the expression of these genes, we analysed 47 genes by the STEM and observed the co-expression of these genes at different growth stages in profile 0 and profile 19 (**Figures 7A, D**). In profile 0, the expression of 22 and 25 genes of Kunlun14 and Menyuanlianglan, respectively, was gradually decreased (**Figures 7B, E**). In profile 19, the expression of 18 genes of Kunlun14 was progressively up-regulated, whereas that of 13 genes of Menyuanlianglan was down-regulated (**Figures 7C, F**).

### 3.6 Identification of lignin and cellulose synthesis pathway genes

**Figure 8:**
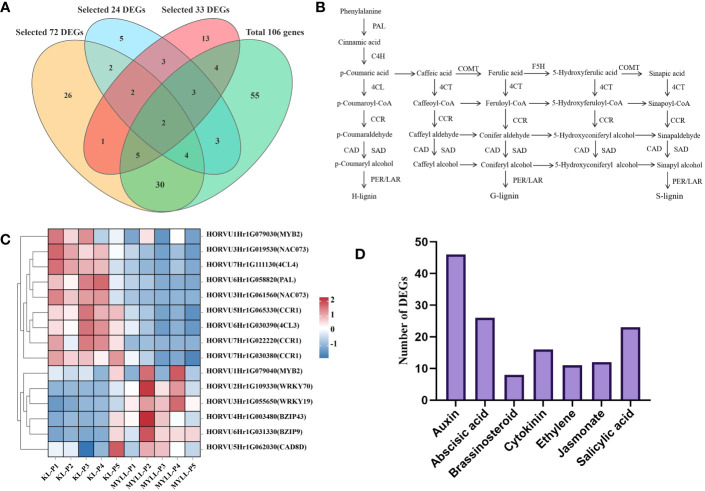
The lignin biosynthesis pathway in hulless barley. **(A)** 17 critical genes associated with lignin, cellulose, and hemicellulose synthesis or regulation. **(B)** Genes involved in this pathway included *PAL*, *PTAL*, *C4H*, *4CL*, *HCT*, *C3H*, *COMT*, *CCoAOMT*, *CCR*, *CAD*, *F5H*, *POD*, and *LAC*. Intermediate metabolites detected in this study with no significant difference between the lodging-resistant and nonresistant groups are marked in blue. Metabolites accumulated in the lodging-resistant group are marked in red. PAL, phenylalanine ammonia-lyase; PTAL/TAL, phenylalanine/tyrosine ammonia-lyase; C4H, cinnamate4-hydroxylase; 4CL, 4-coumarate:coenzyme A ligase; HCT, hydroxycinnamoyl CoA shikimate/quinate hydroxycinnamoyl transferase; C3H, P-coumarate 3-hydroxylase; COMT, caffeic acid O-methyltransferase; CCoAOMT, caffeoyl-CoA O-methyltransferase; CCR, cinnamoyl-CoA reductase; CAD, cinnamyl alcohol dehydrogenase; F5H, ferulate5-hydroxylase; POD, peroxidase; LAC, Laccase. **(C)** Heatmap of 17 key genes related to the synthesis or regulation of lignin, cellulose, and hemicellulose, **(D)** Number of differentially expressed endogenous hormone-regulated genes.

When the DEGs across and within varieties were combined, 17 critical genes associated with lignin, cellulose, and hemicellulose synthesis or regulation (**Table 1**) were identified (**Figure 8A**). Among these DEGs, *PAL*, *4CL3*, *4CL4*, *CCR1*, and *CAD8A* were identified as the biosynthesis pathway genes. PAL is the first enzyme in the phenylpropane metabolic pathway. The activity and expression of PAL can influence the accumulation of lignin and the response of plants to environmental stress. Simultaneously, we discovered several cellulose and hemicellulose biosynthesis genes associated with stem strength, such as cellulose synthase (*CESA4, CESA7*, and *CESA9*), cellulose synthase-like (*CSLH1, CSLC7*, and *CSLA11*), and glycosyltransferases family (*IRX15-L*) (**Figure 8B**).

**Table 1:** The 17 critical genes related to the synthesis or regulation of lignin, cellulose, and hemicellulose.

Type	Gene Symbol	Gene ID	Description
monolignol biosynthesis	*PAL*	*HORVU6Hr1G058820*	phenylalanine ammonia-lyase
	*4CL4*	*HORVU7Hr1G111130*	4-coumarate: Co A ligase
	*CCR1*	*HORVU7Hr1G030380*	cinnamyl-Co A reductase
	*CCR1*	*HORVU7Hr1G022220*	cinnamyl-Co A reductase
	*CAD8A*	*HORVU0Hr1G012940*	cinnamyl alcohol dehydrogenase
cellulose biosynthesis	*CESA4*	*HORVU3Hr1G071770*	Cellulose synthase4
	*CESA7*	*HORVU1Hr1G039250*	Cellulose synthase7
	*CESA9*	*HORVU5Hr1G064230*	Cellulose synthase9
	*CSLA11*	*HORVU3Hr1G109930*	Cellulose synthase-like
	*CSLC7*	*HORVU1Hr1G077230*	Cellulose synthase-like
	*CSLH1*	*HORVU2Hr1G074960*	Cellulose synthase-like
hemicellulose biosynthesis	*IRX15-L*	*HORVU2Hr1G113560*	glycosyltransferases family
TF regulation	NAC100	HORVU2Hr1G017350	NAC transcription factor
	MYB2	HORVU1Hr1G079030	MYB transcription factor
	MYB2	HORVU1Hr1G079040	MYB transcription factor
	WRKY19	HORVU6Hr1G051860	WRKY transcription factor
	WRKY57	HORVU0Hr1G003340	WRKY transcription factor

The expression level of transcription factors such as NAC, MYB, and WRKY was considerably higher in Kunlun14 than in Menyuanlianglan, and the expression of regulatory genes was primarily focussed in the P3-P5 stage. The genes involved in lignin synthesis, on the other hand, were substantially expressed in the P1 and P2 stages in both lodging-resistant and -sensitive varieties. The expression level of these lignin synthesis-related genes decreased in the latter stage (**Figure 8C**).

### 3.7 DEGs associated with phytohormone biosynthesis

Phytohormones are naturally existing small organic signalling molecules that play vital roles in coordinating responses to environmental cues with developmental programmes at vanishingly low concentrations and are indispensable for stress resistance and crop yield (Altmann et al., 2020). The results of KEGG enrichment pathway analysis also showed significant enrichment in plant hormone signal transduction (**Figure 4B**). Many plant hormones including auxin, ethylene, jasmonate (JA), strigolactone (SL), and gibberellin (GA) were dependent on the phenylalanine pathway, tryptophan pathways, etc. (Dong and Lin, 2020). The starting position of the lignin synthesis pathway is also phenylalanine; therefore, we counted the number DEGs involved in the biosynthesis pathways of different hormones. A total of 144 DEGs belonging to the hormone synthesis pathway were identified. Of these, 46 DEGs were most significantly enriched in the auxin biosynthesis pathway, whereas the number of DEGs most significantly enriched in the GA biosynthesis pathway was the least (only one) (**Table S2**). These hormone synthesis-related genes may influence lignin production by controlling the phenylpropane pathway (**Figure 8D**).

### 3.8 WGCNA in different varieties

**Figure 9:**
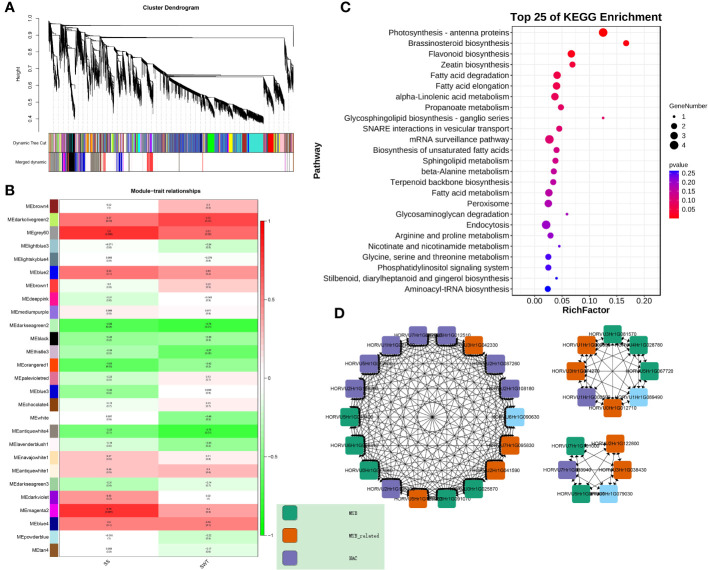
**(A)** Clustering dendrograms of the genes and module detected. **(B)** Correlation analysis between gene expression modules and phenotypes. **(C)** KEGG enrichment analysis in the “grey60”, “magenta2” and “darkolivegreen”“ modules. **(D)** Gene co-expression networks of the “grey60”, “magenta2” and “darkolivegreen” modules.

In order to better analyse the mechanism of lodging resistance, WGCNA was performed on 5 developing samples of Kunlun 14 and Mengyuanlianglan. The dynamic shearing technique was used to cluster the genes and partition them into modules, after which the module vector was calculated, and related modules were merged. In this study, we selected 13011 genes and two traits related to lodging resistance: stem strength, stem wall thickness, and performed WGCNA analysis again (**Figure 9A**). And three models (grey60, magenta2, darkolivegreen2) that may be involved in lodging resistance related traits were obtained (**Figure 9B**). According to KEGG enrichment analysis of the modules, the primary terms enriched by these genes in pathway of flavonoid biosynthesis (It shares a common pathway with lignin synthesis), brassinosteroid biosynthesis (**Figure 9C**). Furthermore, the hub genes associated with lignin production inside the module were screened. We studied the hub genes of the top 20 in two varieties and found many genes related to lignin synthesis. 10 MYB transcription factor, 9 MYB-like transcription factors, and 10 NAC transcription factors were identified as the key genes in the network (**Figure 9D**). These transcription factors may be involved in the regulation of lignin synthesis and other related processes.

### 3.9 Validation of DEGs by qRT-PCR

**Figure 10:**
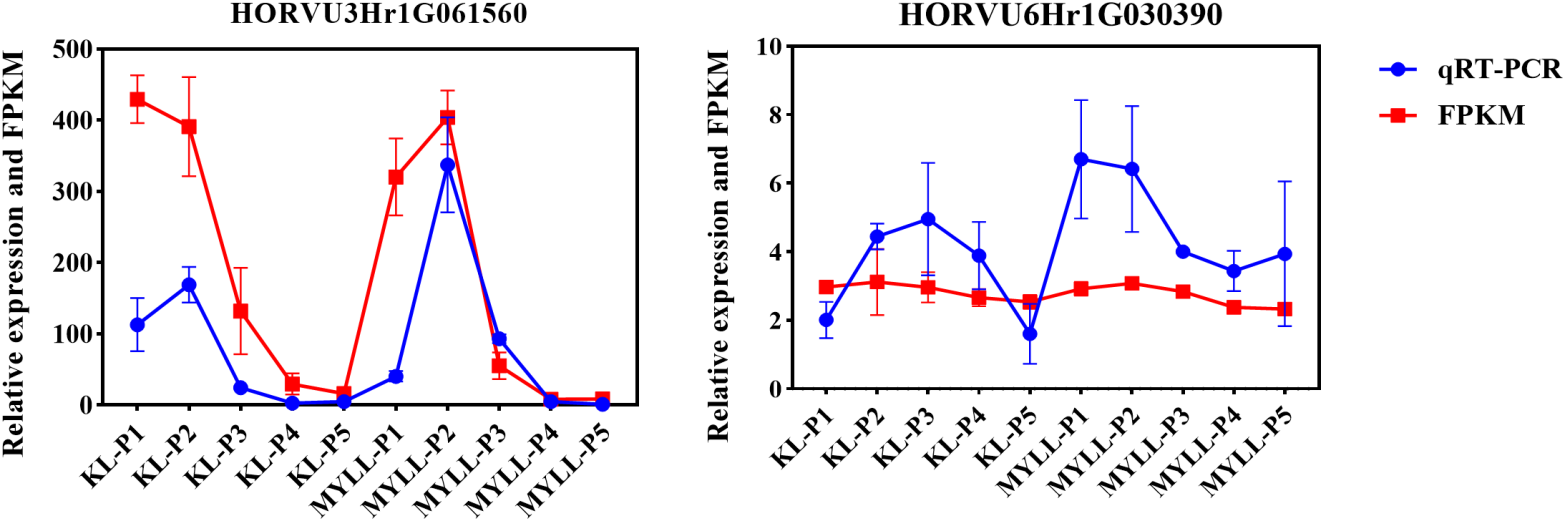
qRT-PCR analysis of two genes for the validation of RNA-seq data.

To confirm the RNA-seq-based gene expression profile, 2 randomly selected DEGs implicated in the lignin, cellulose, and hemicellulose synthesis pathways and regulator genes were chosen for qRT-PCR analysis (**Figure 10**). For normalisation, specific primers were synthesised, and the *Actin* gene was employed as the endogenous control. This validated the RNA-seq results for subsequent data interpretations in this investigation.

## 4 Discussion

Qingke is used not only as a food resource but also as an excellent pasture. It has sparked widespread interest because it contains soluble dietary β-glucans and arabinoxylan, both of which are beneficial to human health (Xu, 1982; Edney et al., 1992). Lignin has long been considered to be inert in the animal digestive tract; hence, it is generally regarded as trivial in animal production, and often, it acts as a component of dietary insoluble fibre to promote gastrointestinal motility (Fahey and Jung, 1983). However, various studies have shown that lignin has various biological activities such as antitumor, antibacterial, antiviral, antioxidant, and anti-inflammatory, indicating that it has a high application value and that its use in animal production should be promoted (Shu et al., 2021).

Lignin, cellulose, and hemicellulose are linked to increased mechanical strength and lodging resistance in stalks (Lewis and Yamamoto, 1990; Cosgrove, 2005). However, the crop is currently facing the problem of lodging. In Qingke, the lignin concentration is shown to be positively correlated to the stem strength (Yu et al., 2021). In the current study, the G-lignin and S-lignin levels were found to be markedly higher in lodging-resistant varieties than in lodging-sensitive types, indicating that G-lignin and S-lignin both play an essential role in increasing the stem mechanical strength of Qingke (Zhao, 2016). Notably, compared with G- and H-lignin, S-lignin has the most recent peak and the most complex molecular structure. The S-lignin concentration increases rapidly during the whole developmental period in the lodging-resistant variety (**Figure 1**). It was hypothesised that S-type lignin contributed significantly to the stem strength of Qingke.

**Figure 11:**
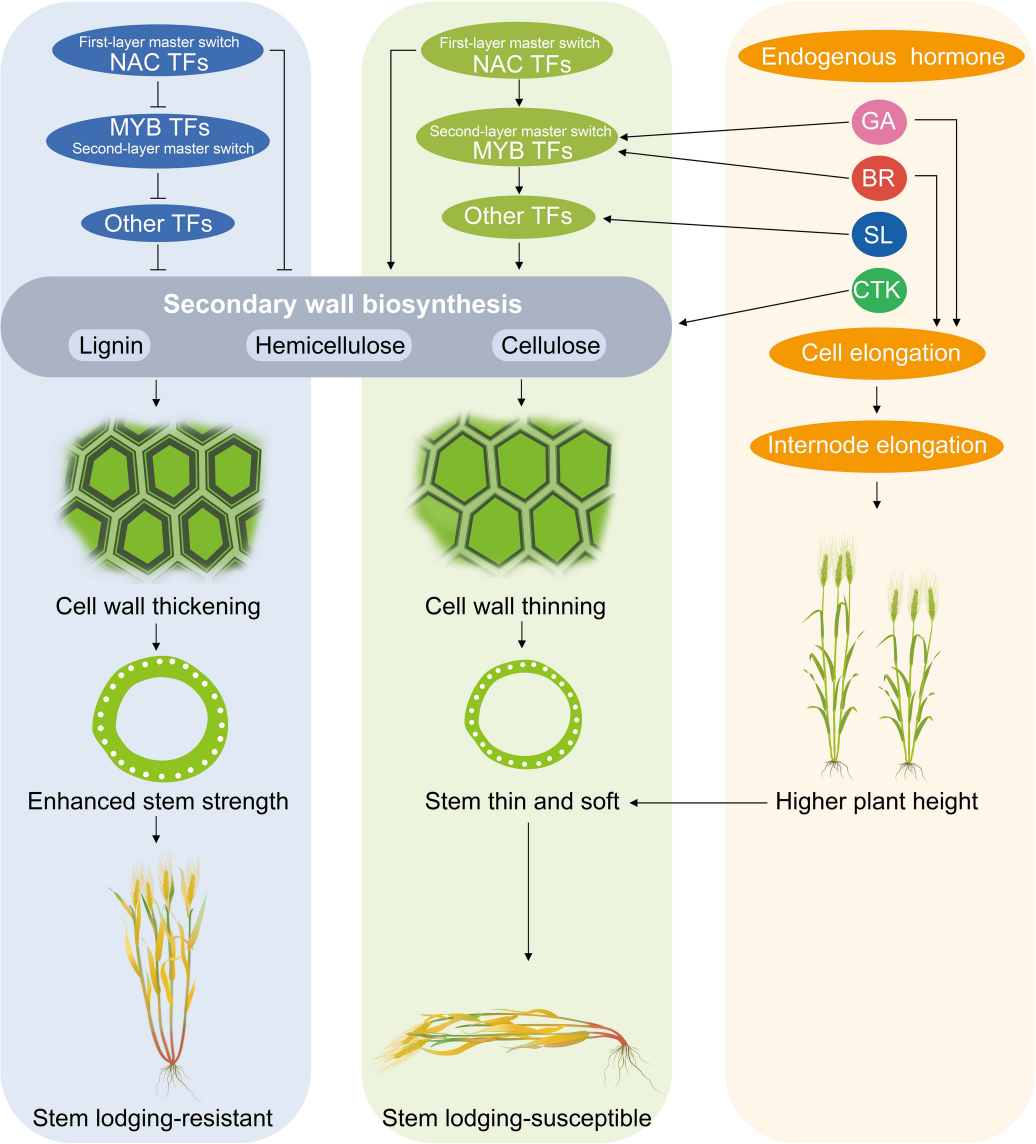
A hypothetic model in this study.

Transcriptome analysis and WGCNA is an efficient method to reveal the correlation between gene expression and phenotypic traits (El-Sharkawy et al., 2015; Zhou et al., 2023). In the present study, we examined the expression of genes involved in the lignin metabolism pathway of hulless barley at different growth and development stages in the two lodging-resistant and -sensitive varieties, Kunlun14 and Menyuanlianglan, through transcriptome sequencing. A total of 17 essential genes or regulatory genes for lignin, cellulose, and hemicellulose synthesis were screened. Lignin biosynthesis originates from the phenylalanine pathway and involves a series of enzyme-catalyzed reactions to produce lignin monomers. The phenylalanine pathway is also a prerequisite for the synthesis of several secondary metabolites, such as anthocyanins, coumarins and flavonoids (Dong and Lin, 2020; Zhou et al., 2022). In this study, both GO and KEGG pathways were enriched for processes related to flavonoid and phenylalanine metabolism (**Figures 2B, C**). In the stems of barley, genes related to lignin, cellulose, and hemicellulose synthesis pathways become gradually inactive or even decline over time (**Figures 3**, **4**). This phenotype is observed in both Kunlun14 and Menyuanlianglan cultivars. However, in the Kunlin14, genes that are continuously up-regulated are still enriched for “secondary metabolic pathways” (**Figure 3**), suggesting that more lignin may be synthesized in Kunlun14, which could be one of the reasons for its lodging resistance. At the same time, our WGCNA results obtained many hub genes related to lignin cellulose, and hemicellulose synthesis. In order to better illustrate our conclusions, we created a schematic diagram (**Figure 11**). The results of this study demonstrate that lodging resistance is influenced not only by synthetic genes and transcription factors but also by the regulation of hormones, which is equally crucial.

In our study, we identified various biosynthesis pathway genes such as PAL, 4CL3, 4CL4, CCR1, and CAD8A, CESA4, CESA7, CESA9, and IRX15-L (**Table 1**). Up-regulation of PAL gene expression in tobacco (Silva et al., 2018), CAD2 and CAD3 in oriental melon (Liu et al., 2020), and 4CL7 in cotton lead to lignin accumulation, which helps plants fight against drought stress. The transcriptional upregulation of lignin biosynthesis genes, such as C4H, C3H, CAD, F5H, HCT, 4CL, COMT, CCR, and CCoAOMT, results in the deposition of lignin, thickening of the secondary cell wall, and enhanced salt and osmotic resistance in white birch (*Betula platyphylla*) and apple (*Malus domestica*) (Chen et al., 2019; Hu et al., 2019). Inhibition of 4CL gene expression in poplar resulted in a sharp decrease in the stem lignin content (Hu et al., 1999), whereas inhibition of CAD gene expression in poplar resulted in a decrease in the S-type lignin monomer content (Barnes and Anderson, 2018). In tobacco, with the decrease in CCR gene expression, lignin in transgenic tobacco decreased both in quality and quantity (Piquemal et al., 1998).

MYB, NAC, and WRKY are important regulatory factors in the lignin synthesis pathway (Gallego-Giraldo et al., 2016; Xiao et al., 2018; Hu et al., 2019; Lin et al., 2023; Ning et al., 2023; Tang et al., 2023). Tamagnone et al. (Tamagnone et al., 1998) first confirmed that the R2R3-MYB transcription factor binds to the AC element and regulates the biosynthesis of lignin. R2R3-MYB transcription factors are a class of plant-specific transcription factors that regulate the expression of structural genes involved in anthocyanin, flavonoid and monolignol biosynthesis (Pratyusha and Sarada, 2022). Several MYB genes have been demonstrated to affect the expression of lignin biosynthetic genes when overexpressed (Zhou et al., 2009). NAC genes are a family of plant-specific transcription factors that regulate the expression of genes involved in stress responses, growth, and secondary wall biosynthesis (Xiao et al., 2018). In Arabidopsis, a series of NAC genes, such as AtNST1, AtNST2, AtSND1 (AtNST3), AtVND6 and AtVND7 played an important role in secondary cell wall synthesis, including lignin, cellulose and xylan biosynthesis (Mitsuda et al., 2005; Zhong et al., 2006; Zhong et al., 2007; Yamaguchi et al., 2008). WRKY genes play an important role in cell wall synthesis. WRKY genes are a family of transcription factors that regulate various plant processes, including stress responses, development, and secondary metabolism (Wang et al., 2010; Feng et al., 2022). WRKY genes can affect secondary cell wall thickening by binding to the promoters of other transcription factors that activate secondary wall synthesis (Wang et al., 2010). Therefore, the five transcription factors obtained in this study may be involved in the synthesis and regulation of lignin, and their specific functions need to be further studied.

In this study, we analyzed comparative transcriptomes both between and within cultivars, and integrated them to obtain a set of key genes involved in the synthesis and regulation of lignin, cellulose, and hemicellulose. DEGs expression analysis revealed that five of these three transcription factors were strongly expressed in the lodging-resistant cultivar Kunlun14 (**Figure 8C**). Further investigation verified whether the DEGs activated the expression of structural genes, such as those related to lignin synthesis. Simultaneously, this study discovered numerous genes related to hormone synthesis. In conclusion, the results providing genes resource for lodging resistance breeding and insight into lodging resistance mechanism.

## Data availability statement

The datasets presented in this study can be found in online repositories. The names of the repository/repositories and accession number(s) can be found below: NCBI BioProject, accession number: PRJNA1014924.

## Author contributions

YB and XZ: investigation, data curation, and manuscript writing. YY: Transcriptome data analyses. XY: WGCNA. LH: lignin content and lignin monomer content determination. XL: GO enrichment analysis. LA: DEG screening and validation of DEGs. KW and ZW: conceived the study, contributed to the design, and modified the draft. All authors contributed to the article and approved the submitted version.
